# 3D transesophageal echocardiography is a decision-making tool for the management of cardiogenic shock following a large postinfarction ventricular defect

**DOI:** 10.1186/s13019-015-0208-y

**Published:** 2015-01-21

**Authors:** Yihua Liu, Zied Frikha, Pablo Maureira, Bruno Levy, Christine Selton-Suty, Jean-pierre Villemot, Olivier Huttin

**Affiliations:** 1 Department of cardiovascular surgery and heart transplantation, 1, Allée du Morvan, F-54500 Vandoeuvre-lès-Nancy, France; 2 Department of cardiology, CHU-Nancy, F-54000 France; 3 Department of critical care medicine, CHU-Nancy, F-54000 France

**Keywords:** Postinfarction ventricular septal defect, 3-Dimensional transesophageal echocardiography, Percutaneous closure, Extracorporeal membrane oxygenation

## Abstract

Postinfarction ventricular septal defect (PIVSD) is a devastating mechanical complication following acute myocardial infarction. The management of this pathology is quite challenging, especially in case of complicated cardiogenic shock. The difficulties lie in the timing and type of intervention. Debates exist with regard to immediate versus deferring repair, as well as open repair versus percutaneous closure. The anatomic characteristics and hemodynamic consequence of PIVSD are important elements determining which strategy to adopt, since large septal defect (>15 mm) cannot be appropriately treated by percutaneous occluder devices limiting by their available size, while compromised hemodynamics usually require emergent repair or mechanical support “bridging to surgery”. Herein, we report our experience of successful management of a case of cardiogenic shock complicating large PIVSD (38 mm) by delayed surgical repair bridged with Extracorporeal Membrane Oxygenation (ECMO) during 7 days. We emphasize the importance of 3-dimensional transesophageal echocardiography as a decision-making tool.

## Background

Postinfarction ventricular septal defect (PIVSD) is a life-threatening mechanical complication of acute myocardial infarction with a declining incidence but a poor prognosis. Its diagnosis and management remain challenging for medical and surgical cardiologic team particularly in the context of cardiogenic shock. The classic dilemma of the management of PIVSD is the timing of intervention: most of the patients require an emergent repair to improve hemodynamics, while intentional deferment of intervention allows reducing the risk of residual shunt through organization and fibrosis of the frail infarct tissue. The evolving percutaneous closure technique is attractive in this critical condition, which can be performed as an alternative of or bridge to surgical repair. However, the percutaneous procedure is generally considered unsuitable in case of large VSD (>15 mm). Hence, a thorough investigation of the anatomical characteristics and hemodynamic impact is mandatory for the management of PIVSD. Real-time three-dimensional transesophageal echocardiography (3-D TEE) provides with comprehensive information on the structural (location, size) and functional (Qp/Qs) parameters of VSD, thus it’s essential for decision-making. Herein, we report our experience in the management of a large PIVSD (38 × 27 mm) which was bridged to successful surgical repair with pharmacological and mechanical support, with emphasis on the therapy-guiding role of the 3-D TEE.

## Case presentation

**Figure 1:**
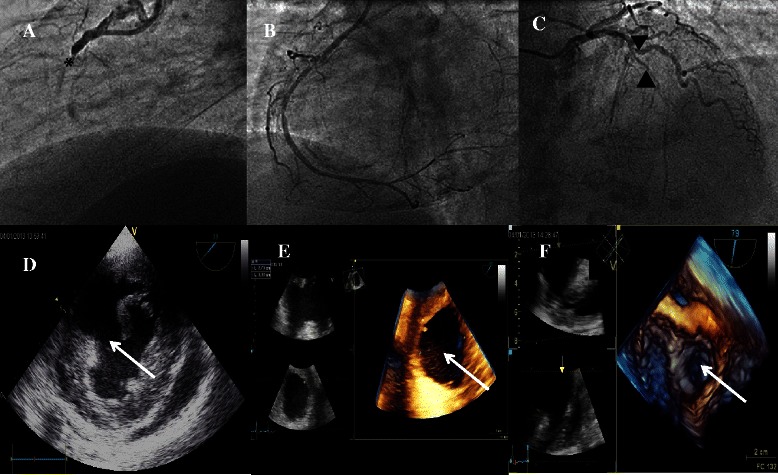
**Preoperative imaging. (A)** Coronary angiography revealed an acute occlusion (*) of the right coronary artery; **(B)** revascularization with percutaneous coronary intervention; **(C)** chronic sub-occlusive lesion of the left anterior descending artery (▲) and a 90% stenosis in the ostium of the first diagonal artery (▼) were also revealed; **(D)** transesophageal echocardiography showed a muscular ventricular septal defect (VSD); **(E)** real-time 3-D transesophageal echocardiography depicted a large septal defect measured 38×27mm; **(F)** the defect was further located in the anterior middle portion of the muscular ventricular septum in the reconstructed image with a view from the left ventricle. The white arrows indicate the location of the VSD.

A 54-year-old man with a family history of coronary artery disease was admitted to our intensive coronary care unit for inferior ST-elevation acute myocardial infarction. The patient was scheduled for emergent primary percutaneous coronary intervention 2 hours following the onset of chest pain. Coronary angiography revealed total occlusion of the right coronary artery (Figure 1A), a chronic subocclusive stenosis in the left anterior descending artery (Figure 1C), and a 90% stenosis in the ostium of the first diagonal artery. Thrombus aspiration and stenting of the culprit right coronary artery was performed with good angiographic results (Figure 1B). Transthoracic echocardiography showed akinetic inferior and inferoseptal wall with an estimated left ventricular ejection fraction of 50%. Right ventricle (RV) was mildly dilated with severe dysfunction (TAPSE 11 mm and S wave tricuspid annulus velocity 9 cm/s). Neither mitral regurgitation nor pericardial effusion was reported.

Three days after hospital admission, the patient complained of dyspnea and developed cardiogenic shock. Physical examination revealed a newly onset grade III/VI holosystolic murmur audible throughout the precordium. The hemodynamics continued to deteriorate despite inotropic support, which justified a mechanical circulation support with peripheral (femoral) veno-arterial extracorporeal membrane oxygenation (ECMO). The hemodynamic parameters were thereafter stabilized. Another transthoracic echocardiography with Doppler color flow revealed a high-velocity left-to-right ventricular shunt suggesting a PIVSD (Figure 1D). Poor quality of images obtained with parasternal view rendered them uninterpretable in this recumbent patient with ECMO. An emergent thansesophageal echocardiography (TEE) was therefore necessary. The two-dimensional (2D) TEE identified a large VSD (Figure 1D) and a mild mitral regurgitation, Qp/Qs was calculated to be 2.8. As the initial 2D morphological findings were insufficient to comprehend the relationship between the defect and the surrounding structures, we performed real-time three-dimensional (3D) TEE images to acquire detailed findings with regard to the size, the shape and the relationship with surrounding tissue, which confirmed an anterial muscular septal defect measuring 27*38 mm (Figure 1E, F), and revealed a mild mitral valve regurgitation, without papillary muscle tearing nor mitral chordae rupture being detected.

**Figure 2:**
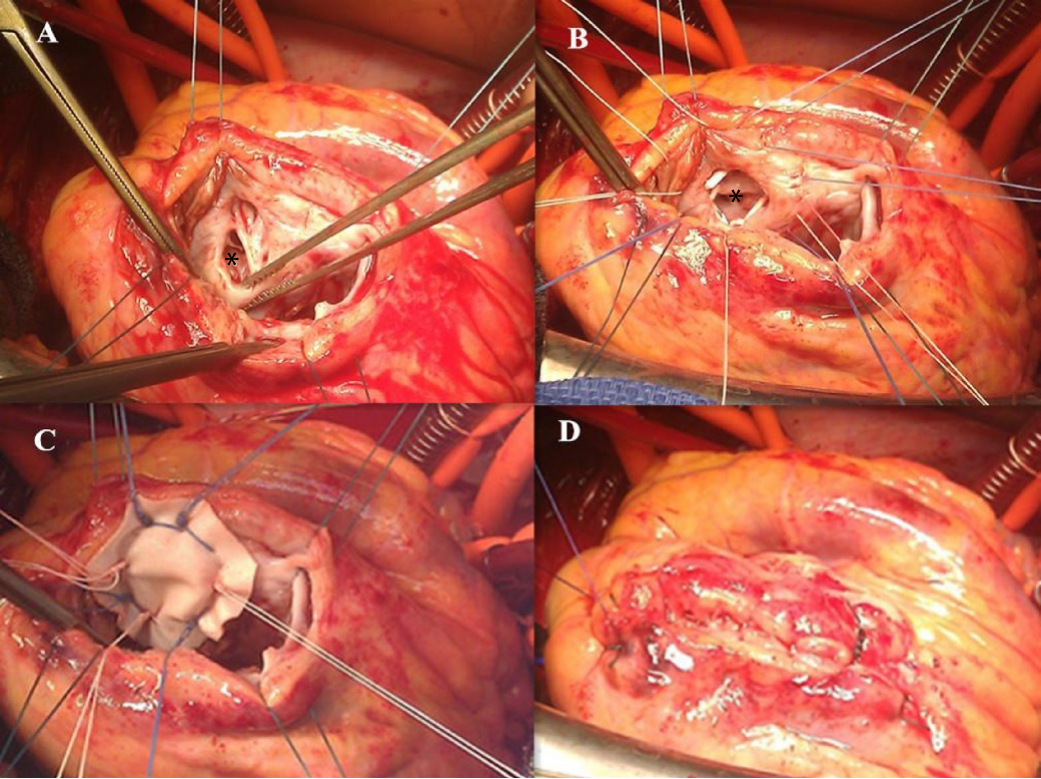
**Per-operative photos. (A)** the septal defect (*) was approached by a left ventricular transinfarct\incision; **(B)** a series of pledgeted sutures were placed around the rim of septal defect which seemed to be firm enough; **(C)** the septal defect was repaired with a Dacron patch; **(D)** closure of the transfarct incision.

As the hemodynamics was maintained with ECMO, a semi-elective operation was performed 7 days following initial mechanical support. After median sternotomy, ECMO was temporarily ceased, femoral venous cannula was retrieved back for 10 cm, and cardiopulmonary bypass was instituted with bicaval cannulation. The VSD was approached by the left ventricular transinfarct incision (Figure 2A). Consistent with TEE finding, intraoperative exploration revealed a large anterior VSD of approximately 4 cm in major diameter, and the rim of septal defect appeared to be consolidated by the fibrous scar (Figure 2B). The VSD was repaired (Figure 2C, 2D) with a Dacron patch (Hemashield Finess, Boston Scientific, Boston, MA) using interrupted 2–0 Ethibond pledgeted sutures. Cardiopulmonary bypass was weaned with a high dose of inotropes and the ECMO flow was resumed. Post-operative echocardiography excluded residual left-to-right shunt and mitral regurgitation. Postoperative evolution was favorable with rapid resolution of cardiogenic shock situation. The patient was weaned from ECMO and inotropic agents on postoperative day 15. The recovery was uneventful and the patient was doing well 6 months later with NYHA class II.

## Discussion

Post-infarction VSD remains an infrequent but devastating complication of atherosclerotic coronary disease. The incidence of this complication has significantly decreased to < 1% of cases with the advent of early reperfusion strategies and adjunct medical therapy [1]. Data on the impact of primary percutaneous coronary intervention and the incidence of VSD are limited. According to Yip et al. [2] primary PCI has a significant impact on the incidence of this complication. The prognosis of post-AMI VSD is very poor, with mortality rates reaching 47% even with intervention in the elderly population [3]. Furthermore, complex forms of ventricular septal rupture with right ventricle involvement and onset of cardiogenic shock are critical prognostic factors [4].

When a ventricular septal rupture complicating acute myocardial infarction is suspected, transthoracic and/or transesophageal echocardiography at patient bedside is the test of choice for early diagnosis and therapy guidance. For this purpose, unconventional transthoracic and subcostal echocardiographic views with color flow doppler mapping to detect the site of septal rupture and visualize right ventricular free wall are mandatory. Unfortunately the acquisition of quality images is challenging and often impossible in recumbent haemodynamically unstable patients with mechanical support. The lack of parasternal window in our case triggered further difficulties for accurate transthoracic echocardiographic diagnosis. 2D TEE allows visualization of the ventricular septum from multiple orthogonal planes; however, it requires a mental 3D reconstruction to better understand the relationship between the defect and the surrounding structures. The introduction of three-dimensional echocardiography offers new imaging possibilities with precise localization and easier definition of the defect anatomy. It provides unique en-face views of the ventricular septum from both left and right ventricular sides [5]. New developments of the 3D matrix array probe allow real-time 3D imaging with high resolution. So, 3D TEE offers additional special information in VSD disease without extending examination time, permits quantitative recording of septal defect dynamics and enhances the understanding of complex cardiac anatomy and hemodynamics. It is a potentially valuable clinical tool for diagnosing and managing patients with VSD [6]. Cheng et al. [7] reported an excellent correlation in measuring the size of VSD by 3D echocardiography compared with intraoperative evaluation.

The management of PIVSD consists of surgical repair and/or percutaneous closure. Limited by the size with the largest device available being 24-mm in diameter, the percutaneous occluder closure is attractive in conditions of small VSD (<15 mm) and/or patients being poor surgical candidate [8]. Sporadic case reports suggested that percutaneous occluder devices could be implanted for the purpose of bridging to surgical repair [9]. Nevertheless, the fragility of infarct myocardium in acute setting is the major challenge for open repair and transcatheter closure. Even though the timing for intervention is a debating issue, it’s generally accepted that, if the patient’s status allows, intentional deferment of intervention by 3 to 4 weeks will facilitate surgical repair and reduce the risk of residual shunt by tissue healing. In our case, the size of VSD (38 mm) measured by 3D TEE excluded the feasibility of percutaneous closure procedure. Hence, we adopted the strategy of mechanical support prior to and following surgical repair. Pre-operative mechanical support allowed stabilizing the patient’s hemodynamics, unloading the ventricles and decreasing the left-to-right shunt as well as consolidating the infarct tissue through fibrous scar formation; post-operative mechanical assist facilitated the tissue healing and reduced the risk of residual shunt as ECMO led to ventricular pressure and volume unloading that reduced the wall stress and suture tension.

Our successful experience in the management of this challenging case demonstrates the therapy-guiding values of 3-D TEE in the management of PIVSD, as precise preoperative anatomical assessment of PIVSD is essential to determine the therapeutic strategy. Our algorithm of management of PIVSD is to perform thorough echocardiographic exams at first, if the size < 15 mm and the unfavorable anatomical characteristics such as frail rim tissue and papillary muscle involvement are excluded, percutaneous closure can be thereafter performed as a definitive treatment or bridge to surgical repair; in other cases, pharmacological and/or mechanical support for at least 1 week is adopted before surgical repair.

## Conclusion

Dynamic 3D TEE provides important additional diagnostic information and is a useful technique in the assessment of patients with PIVSD. It enhances the understanding of the anatomy of the lesion and should be an important process in the choice of therapeutic options (device closure or surgical procedures). Combined with 2D techniques, it is highly reliable for the preoperative assessment of PIVSD.

## Consent

Written informed consent was obtained from the patient for publication of this Case report and any accompanying images. A copy of the written consent is available for review by the Editor-in-Chief of this journal.

## Abbreviations

PIVSD: Postinfarction ventricular septal defect
ECMO: Extracorporeal membrane oxygenation
VSD: Ventricular septal defect
3-D TEE: Three-dimensional transesophageal echocardiography

## References

[1] Renshaw BS, Granger CB, Birnbaum Y, Pieper KS, Morris DC, Kleiman NS (2000). Risk factors, angiographic patterns, and outcomes in patients with ventricular septal defect complicating acute myocardial infarction. GUSTO-I (Global Utilization of Streptokinase and TPA for Occluded Coronary Arteries) Trial Investigators. Circulation.

[2] Yip HK, Fang CY, Tsai KT, Chang HW, Yeh KH, Fu M (2004). The potential impact of primary percutaneous coronary intervention on ventricular septal rupture complicating acute myocardial infarction. Chest.

[3] Blanche C, Blanche DA, Denton TA, Khan SS, Kamlot A, Trento A (2000). As originally published in 1994: postinfarction ventricular septal defect in the elderly: analysis and results. Updated in 2000. Ann Thorac Surg.

[4] Vargas-Barron J, Molina-Carrion M, Romero-Cardenas A, Roldan FJ, Medrano GA, Avila-Casado C (2005). Risk factors, echocardiographic patterns, and outcomes in patients with acute ventricular septal rupture during myocardial infarction. Am J Cardiol.

[5] Acar P, Abdel-Massih T, Douste-Blazy MY, Dulac Y, Bonhoeffer P, Sidi D (2002). Assessment of muscular ventricular septal defect closure by transcatheter or surgical approach: a three-dimensional echocardiographic study. Eur J Echocardiography.

[6] Mercer-Rosa L, Seliem MA, Fedec A, Rome J, Rychik J, Gaynor JW (2006). llustration of the additional value of real-time 3-dimensional echocardiography to conventional transthoracic and transesophageal 2-dimensional echocardiography in imaging muscular ventricular septal defects: does this have any impact on individual patient treatment?. J Am Soc Echocardiogr.

[7] Cheng TO, Xie MX, Wang XF, Wang Y, Lu Q (2004). Real-time 3-dimensional echocardiography in assessing atrial and ventricular septal defects: an echocardiographic-surgical correlative study. Am Heart J.

[8] Maltais S, Ibrahim R, Basmadjian AJ, Carrier M, Bouchard D, Cartier R (2009). Postinfarction ventricular septal defects: towards a new treatment algorithm?. Ann Thorac Surg.

[9] Costache VS, Chavanon O, Bouvais H, Blin D (2007). Early Amplatzer occluder closure of a postinfarct ventricualr septal defect as a bridge to surgical procedure. Interact Cardiovasc Thorac Surg.

